# Supplementary figures and images for: The Effects of Active Methamphetamine Use Disorder and Regular Sports Activities on Brain Volume in Adolescents
Source: J Clin Med. 2025 Jul 23;14(15):5212. doi: 10.3390/jcm14155212 (PMC12347424; doi:10.3390/jcm14155212)

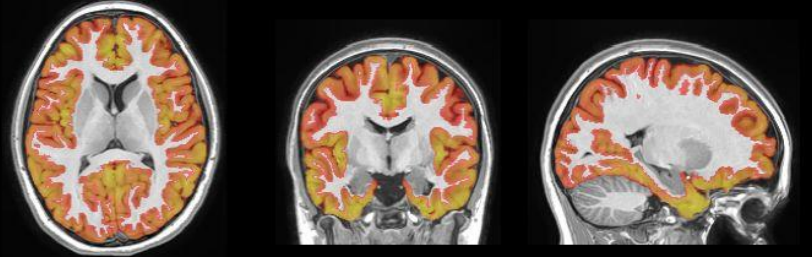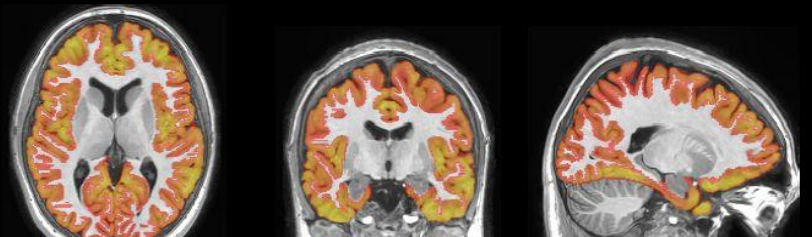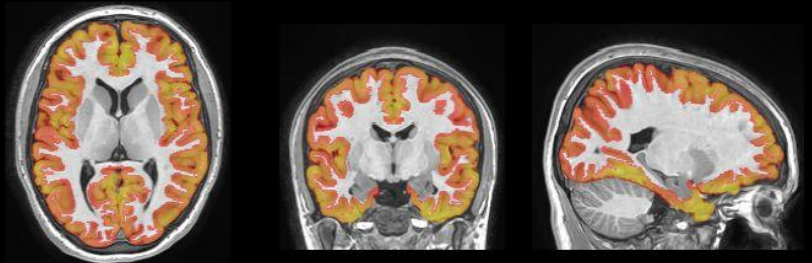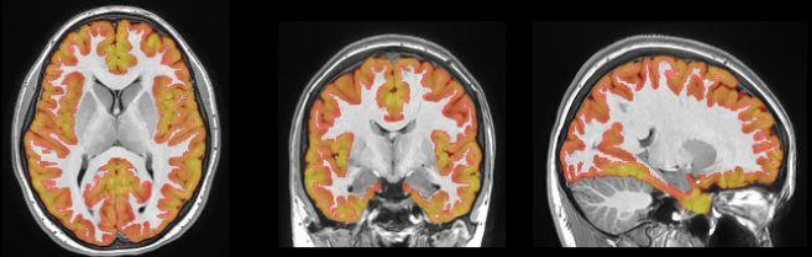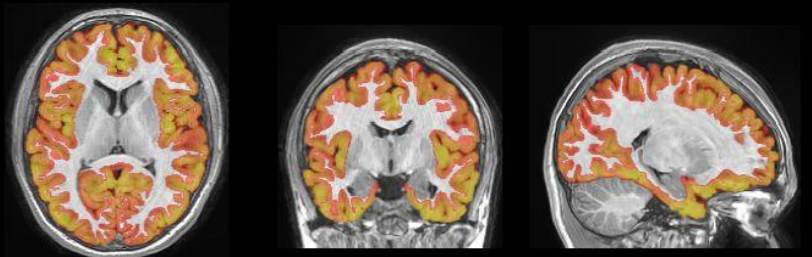

Control

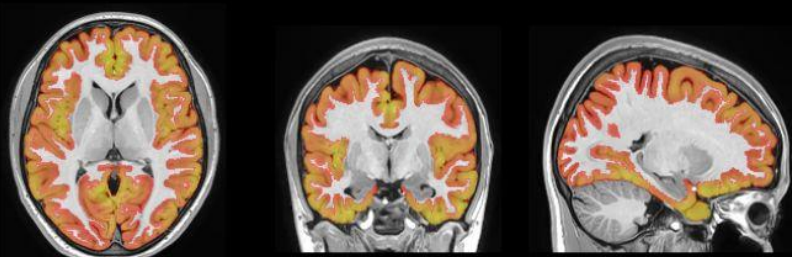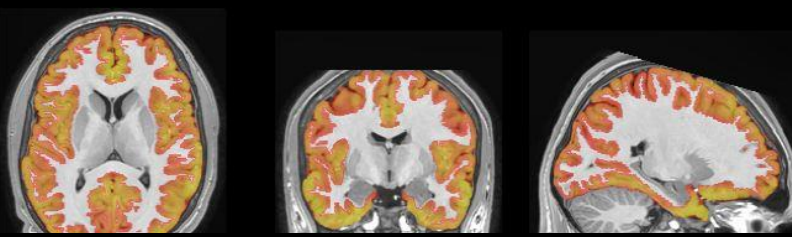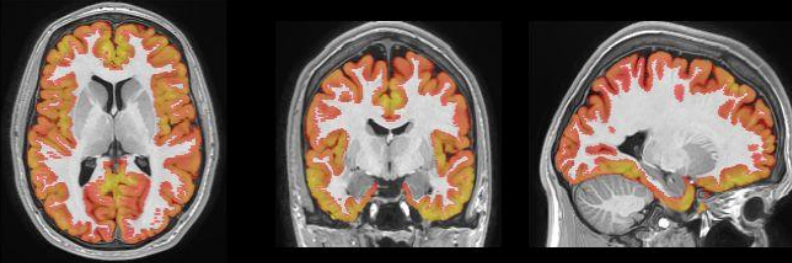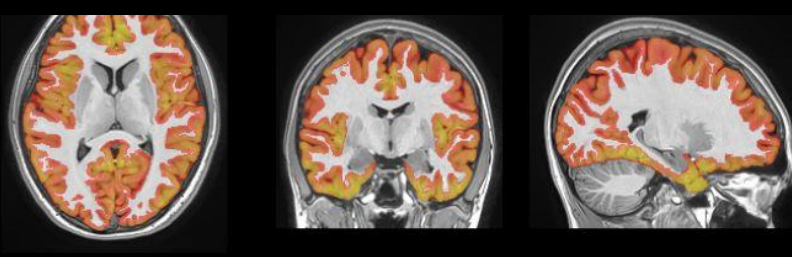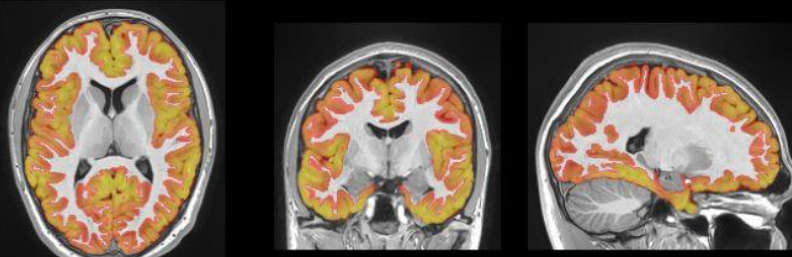

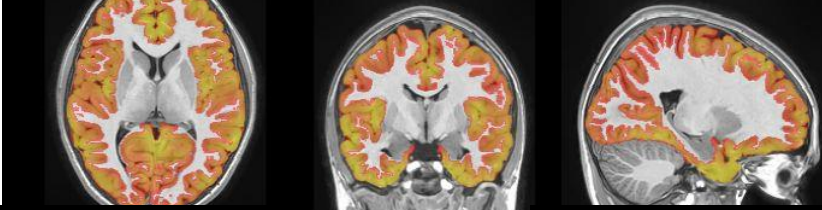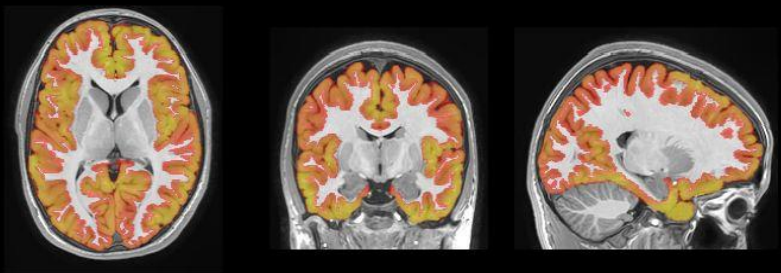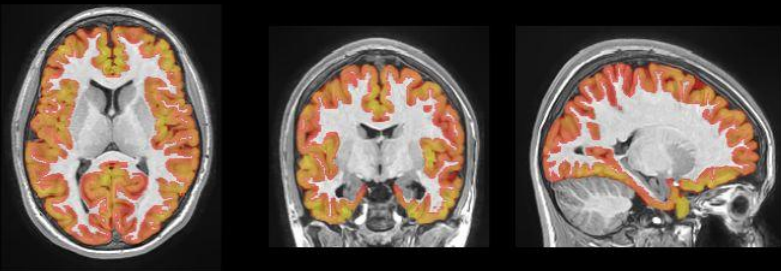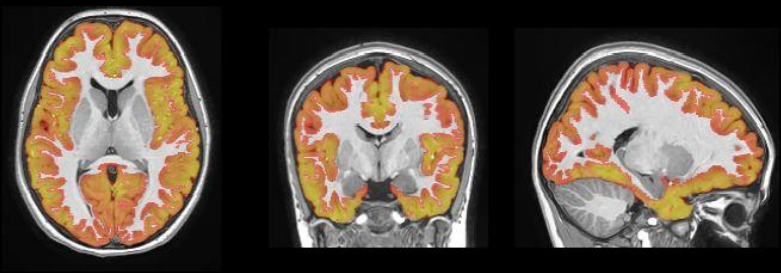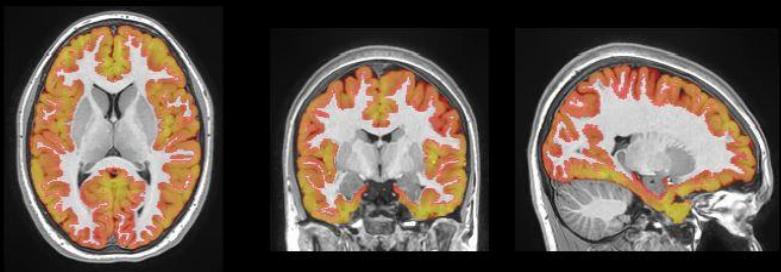

Athletes

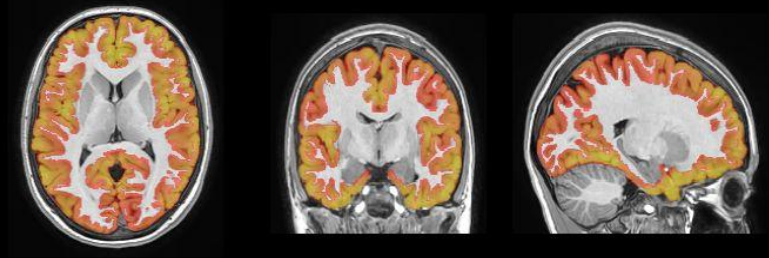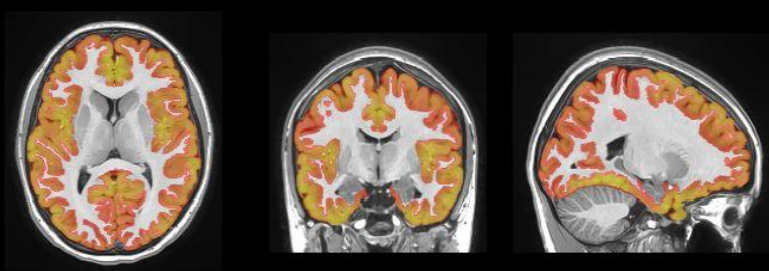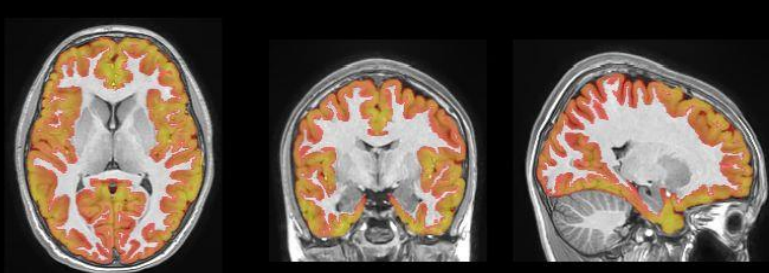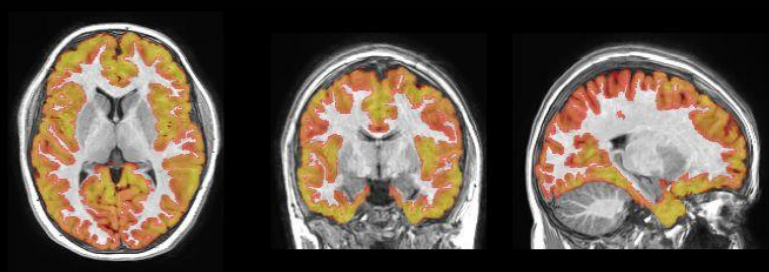

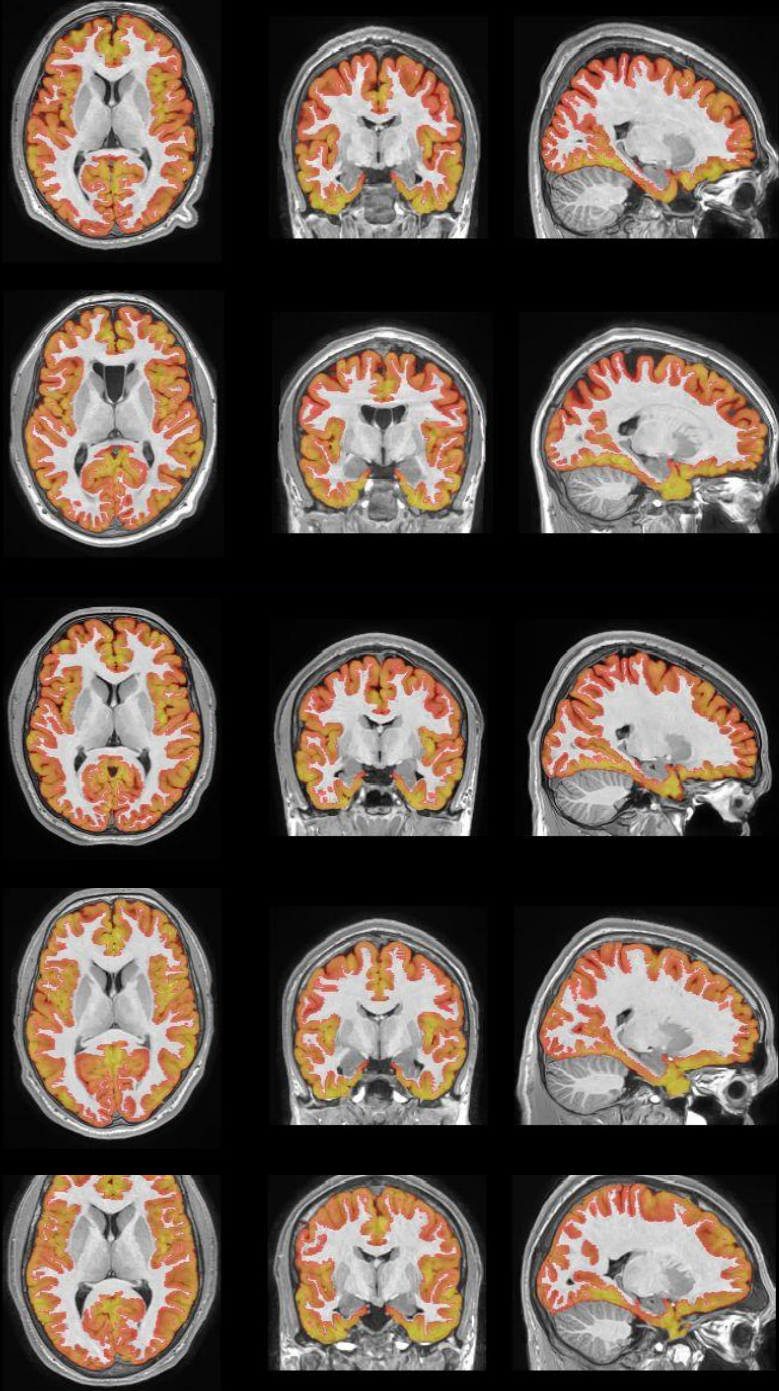

## Adolescents with MUD

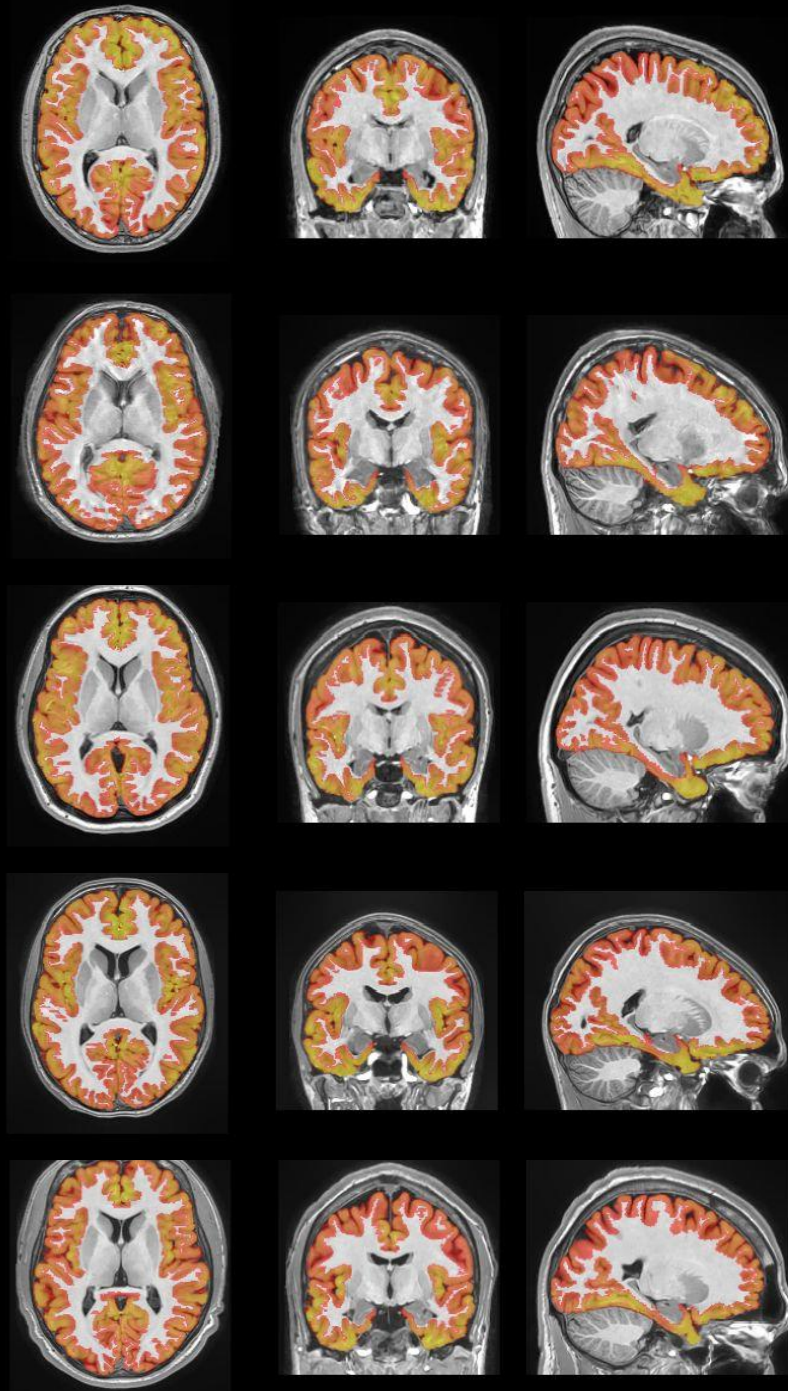

Supplement: Supplementary file 1 [file jcm-14-05212-s001.zip › jcm-3704587-supplementary.pdf]
